# Resilience in adolescence during the COVID-19 crisis in Canada

**DOI:** 10.1186/s12889-023-15813-6

**Published:** 2023-06-06

**Authors:** J. Chin, J. Di Maio, T. Weeraratne, K. M. Kennedy, L. K. Oliver, M. Bouchard, D. Malhotra, J. Habashy, J. Ding, S. Bhopa, S. Strommer, P. Hardy-Johnson, M. Barker, D. M. Sloboda, L. McKerracher

**Affiliations:** 1 Department of Biochemistry and Biomedical Sciences, McMaster University, 1280 Main Street West, HSC 4H30A, HamiltonHamilton, ON L8S 4K1 Canada; 2 Faculty of Dentistry, University of Toronto, Toronto, ON Canada; 3 Farncombe Family Digestive Health Research Institute, McMaster University, Hamilton, ON Canada; 4 Department of Obstetrics and Gynecology, McMaster University, Hamilton, ON Canada; 5 Werklund School of Education, University of Calgary, Calgary, AB Canada; 6 Department of Epidemiology, and Occupational Health, McGill University, BiostatisticsMontreal, QC Canada; 7 Department of Medicine, McMaster University, Hamilton, ON Canada; 8 Faculty of Health Sciences, McMaster University, Hamilton, ON Canada; 9 MRC Lifecourse Epidemiology Centre, University of Southampton, Southampton, UK; 10 Primary Care Population Sciences and Medical Education, Faculty of Medicine, University of Southampton, Southampton, UK; 11 Department of Pediatrics, McMaster University, Hamilton, ON Canada; 12 Department of Public Health, Aarhus Institute for Advanced Studies, Aarhus University, Høegh-Guldbergs Gade 6B, 8000 Aarhus, Denmark

**Keywords:** Adolescence, COVID-19, Health behaviours, Mental health, Mixed methods, Resilience

## Abstract

**Background:**

The COVID-19 pandemic constitutes a social crisis that will have long-term health consequences for much of the global population, especially for adolescents. Adolescents are triply affected as they: 1) are experiencing its immediate, direct effects, 2) will carry forward health habits they develop now into adulthood, and 3) as future parents, will shape the early life health of the next generation. It is therefore imperative to assess how the pandemic is influencing adolescent wellbeing, identify sources of resilience, and outline strategies for attenuating its negative impacts.

**Methods:**

We report the results of longitudinal analyses of qualitative data from 28 focus group discussions (FGDs) with 39 Canadian adolescents and of cross-sectional analyses of survey data from 482 Canadian adolescents gathered between September 2020 and August 2021. FGD participants and survey respondents reported on their: socio-demographic characteristics; mental health and wellbeing before and during the pandemic; pre- and during-pandemic health behaviours; experiences living through a crisis; current perceptions of their school, work, social, media, and governmental environments; and ideas about pandemic coping and mutual aid. We plotted themes emerging from FGDs along a pandemic timeline, noting socio-demographic variations. Following assessment for internal reliability and dimension reduction, quantitative health/wellbeing indicators were analyzed as functions of composite socio-demographic, health-behavioural, and health-environmental indicators.

**Results:**

Our mixed methods analyses indicate that adolescents faced considerable mental and physical health challenges due to the pandemic, and were generally in poorer health than expected in non-crisis times. Nevertheless, some participants showed significantly better outcomes than others, specifically those who: got more exercise; slept better; were food secure; had clearer routines; spent more time in nature, deep in-person social relationships, and leisure; and spent less time on social media.

**Conclusions:**

Support for youth during times of crisis is essential to future population health because adolescence is a period in the life course which shapes the health behaviours, socio-economic capacities, and neurophysiology of these future parents/carers and leaders. Efforts to promote resilience in adolescents should leverage the factors identified above: helping them find structure and senses of purpose through strong social connections, well-supported work and leisure environments, and opportunities to engage with nature.

**Supplementary Information:**

The online version contains supplementary material available at 10.1186/s12889-023-15813-6.

## Background

Socio-environmental disasters including wars, earthquakes, major storms, and infectious disease epidemics radically alter human behaviours and social structures. Such crises negatively impact short-term, longer-term, and even multi-generational health behaviours and outcomes [1]. The COVID-19 crisis is no exception. It will undoubtedly have population-wide health impacts over the entire life course with potential inter-generational effects. On March 11, 2020, the World Health Organization (WHO) declared Coronavirus disease-19 (COVID-19) a pandemic. In response, most national and many regional governments outlined and implemented measures to mitigate the transmission of COVID-19 [2]. The Canadian national, regional, and municipal governments followed WHO recommendations and put extensive mitigation measures in place. Key measures included restricting community movements, closing non-essential businesses, moving education online, and banning all forms of travel [3]. These measures dramatically altered daily life for Canadians of all ages. This drastic change from normal life led to many new stresses and challenges, as well as opportunities.

Some populations are particularly vulnerable to the impacts of upheavals in daily life. Adolescents comprise one of these vulnerable populations. Adolescence is a transitional period between childhood and adulthood that occurs between 10 and 24 years of age, with the teen years (13–19) comprising the biggest biological, neurobiological, and social transitions for most young people. In high income countries including Canada [4] (the focus of the present study), adolescence is a period in the life course when key behavioural habits (e.g. diet, physical activity, sleep, etc.) are formed [5]. During adolescence, the brain regions responsible for cognition, and emotional and behavioural regulation develop, facilitating increased capacities for self-awareness, self-direction, and self-regulation [6]. These biological changes influence adolescent behaviour including identity exploration; development of autonomy, new social skills, and new relationships; initiation of new adulthood-related goal-setting (particularly with respect to education and career paths); and the seeking of increased social and financial independence [7]. Adolescents also experience heightened emotional responses to stressors, which can be difficult to manage as the self-regulatory system develops [6].

Irrespective of the pandemic or other forms of crisis, adolescents generally face a number of physical and mental health vulnerabilities. Most engage in relatively poor health behaviours including exercising insufficiently, sleeping inadequately, and eating nutritionally-poor diets [8–10]. Pre-pandemic, 81% of adolescents globally were physically inactive, with older adolescents exhibiting even lower physical activity levels than their younger counterparts [8]. Generally, adolescent sleep patterns vary across the week, at least in higher-income countries: on school nights, adolescents get less sleep than recommended, whereas on weekends, they get adequate sleep [9]. Insufficient weekday sleep may be attributed to a large amount of screen time combined with school- or work-imposed wake up times – psychological and physical arousal of social media content has been linked to poor sleep [11]. In terms of dietary behaviours, adolescents in Canada rarely meet recommended dietary guidelines, consuming inadequate amounts of fruits and vegetables and typically eating energy-dense, nutrient-poor foods [10].

Pre-pandemic, at least 10–20% of adolescents around the world were already experiencing or had experienced some form of mental illness, although this rate is likely an underestimate because a large proportion of adolescent mental illnesses go undiagnosed [12]. As of 2019, 7% of Canadians aged 12–14 years and 17% of those aged 15–17 years self-reported their mental health was “fair” or “poor”, suggestive of low preparedness for a mental health shock in a non-trivial minority of youth. Adolescents generally tend to use avoidant coping mechanisms including distraction, disengagement, and/or substance use when dealing with psycho-social challenges and transitions. These avoidant tactics provide immediate relief or prevent negative feelings from occurring [13]. While they may be effective in the short term, they are ineffective against chronic stressors and may lead to problematic behavioural patterns and habits and poor adaptation to adversity [14]. The COVID-19 pandemic is an example of a chronic stressor for which many adolescents were unprepared. While increased anxiety and depression levels amongst adolescents have been the most widely cited challenges, other mental health concerns related to the pandemic include increased levels of loneliness, distress, anger, irritability, boredom, fear, stress, hyperactivity, and difficulties concentrating [15, 16].

Despite these risks and vulnerabilities associated with adolescence for the majority of young people, there are also reasons to expect that *some* adolescents are relatively well-prepared to respond to a crisis like the COVID-19 pandemic with strength, optimism, and positive coping strategies. These are young people with relatively more resilience. Resilience refers to the latent and/or learned ability to respond positively and adaptively to adversity – both at an individual or a community/systems level. Resilience is determined by personal (e.g. self esteem or emotional regulation), biological (e.g. changes in brain chemistry caused by neurotransmitter receptor sensitivity), and systemic (e.g. community, culture or family) factors [17], which, in turn, feed into the motivational, socio-emotional, and behavioural factors that shape coping strategies.

Emphasizing opportunities for building resilience, in this mixed methods study, the “CANadian Teens Experiencing COVID-19 (CANTEC-19) Study”, we aimed to explore the impacts of the COVID-19 pandemic on subjective physical and mental wellbeing, health behaviours, and lived experiences in a sample of adolescents living in and/or attending school in a mid-sized urban center in Canada. We hypothesized that restrictions on social interactions would seriously compromise basic adolescent psychological needs for autonomy, competence, and relatedness [18], and therefore adolescents’ capacity to stay well and healthy. We further expected that imposed constraints and barriers on positive coping strategies like group engagement (social, sport, artistic, or teamwork) or leadership activities (paid or unpaid), would impede core adolescent psychological needs, with impacts on coping ability. We also expected that individuals with indicators of greater resilience prior to the pandemic would have relatively better health and wellbeing outcomes during the pandemic. Assuming that factors underlying the capacity to build resilience might be leveraged to develop intervention strategies to support adolescent wellbeing in times of crisis, we aimed to identify factors associated with indicators of resilience.

## Methods

### Participants and setting

CANTEC-19 study participants were adolescents between the ages of 12 and 20 years living or attending school in the City of Hamilton, Ontario, Canada. Participants (and at least one of their parent(s)/legal guardian(s), for those under 18 years of age) had to be capable of understanding the information, assent, and consent forms (i.e. reading and comprehending at a grade 8 level; these forms were only available in English).

Located in Southwestern Ontario, Hamilton is a statistically-average Canadian city in terms of size, age structure, educational attainment levels, percent of recent immigrants, percent of visible minorities, living arrangements, and walkability/commutability [19]. The average age of Hamilton residents is 41.5 years, similar to the Canadian average of 41.7 years. [20] In 2020, the median after-tax income of households in Hamilton was $75,500, which is less than that for the province of Ontario as a whole ($79,500) [21]. Despite being socio-demographically average and thus, in some ways, a reasonable representation of Canada more broadly [19], Hamilton exhibits stark geographical disparities in the determinants of health [20, 22]. Striking differences in poverty and education levels drive vastly different health outcomes (e.g. lower life expectancy, higher emergency room visits) depending on neighbourhood location [20, 22]. With these geographic inequities in mind, we purposefully sought study engagement from adolescents living in different Hamilton neighbourhoods.

### Data collection

We employed a mixed methods, behaviour- and experience-focused sequential exploratory approach, which used quantitative and qualitative survey data and qualitative data collected from focus group discussions (FGDs). Participants were recruited through social media (Facebook, Twitter, Instagram), university message boards, word of mouth, and through the local public school board.

#### Survey

The survey comprised a 98-item online anonymous questionnaire including socio-demographic characteristics, indicators of mental health and wellbeing, general activity patterns, health-related behaviours (workload, diet, physical activity, sleep, personal hygiene, screen time) and constraints/enablers of health behaviours (e.g. food security, special dietary restrictions, availability of social supports, postal code/neighbourhood). Open-ended questions at the end of the survey allowed respondents to share context and narratives about their experiences. The survey was shared via social media and distributed to secondary school parents/guardians via the local public school to some 13,000 eligible students/parents/guardians in the Hamilton public school board. All respondents to the online survey were offered a chance to enter a draw for one of four $100 Amazon gift cards. The online survey link was open from September 2020 to August 2021. Details on each of the variables measured are as follows:

##### Sociodemographic characteristics

Age (years), postal code (first three digits), race/ethnicity (self-reported, check all that apply), and gender (self-report pronouns) were collected via questions based on those in the Canadian Community Health Survey [23]. Postal code prefixes were used to infer respondents’ likely socio-economic position, based on their neighbourhood (see section on “barriers and enablers of health behaviours” for further details).

##### Mental health and wellbeing

Participant mental health and wellbeing was assessed via a series of three validated scale-based instruments: a 14-item Short Warwick-Edinburgh Mental Wellbeing Scale (SWEMWS; hereafter referred to as “resilience”) [24]; two Cantril Ladders (hereafter “pre-pandemic life satisfaction” and “current [i.e., time of survey response] life satisfaction) [25]; and a 9-item Child Health Utility 9D (CHU9D; hereafter “mental distress”) Questionnaire [26]. These are all measures of psychological wellbeing that have been validated for use with youth [24–26]. As indicated by their Cronbach's alpha values over 0.8, both the resilience scale (SWEMWS) and the mental distress scale (CHU9D) have robust internal construct validity in this sample. Additional details regarding scoring of these mental health and wellbeing scales/ladders are available in supplementary materials (See Supplementary File).

##### Activity patterns, health-related behaviours and diet

Respondents completed a series of questions regarding their activity patterns, including questions about their sleep, screen time, physical activities/exercise, engagement in hobbies, social interaction, and personal hygiene practices. These indicators were each assessed using activity frequency scales, and were scored between 0 which indicated never or very rarely engaging in a given activity or facing a given disruption/challenge and 4, 5, or 6 (depending on the scale) which indicated frequently engaging in that activity or facing that challenge. Details of the individual activity scale items are in supplementary materials (See Supplementary File). To assess overall diet quality, respondents completed an 18-item food frequency questionnaire (FFQ)–a Canadian-adapted version [27] of the PrimeScreen short dietary assessment tool [28], which we refined slightly (listed additional food category example foods for most categories) to reflect Canada’s increasing cultural diversity. To make the survey experience more appealing to Generation Z adolescents, a highly-visual population of digital natives [29], we also included pictures of example foods from each food category.

##### Barriers and enablers of health behaviours

To assess food security, respondents were asked two questions comprising a validated, 2-item truncated food security screener [30] about their ability to access food/groceries over the previous 12 months. The first three digits of respondents’ postal codes identified the location of residence of respondents at the level of the neighbourhood. Using postal code prefix maps of Hamilton as presented in the Hamilton Community Foundation’s *Vital Signs* (2015) report, postal code prefixes were coded according to the prevalence of children living in low income households (as of 2009): over 33% (high), between 18–32% (medium), or under 18% (low) [31]. For the purposes of our analyses, we combined postal code prefixes with medium and high prevalence of low income households together and assigned them a dummy score of 1 (higher child poverty). We also combined postal code prefixes with lower rates of low income households with those who reported attending school in Hamilton but living outside of its postal code boundaries. This group was assigned a dummy score of 0 (lower child poverty).

#### Focus Group Discussions (FGDs)

Focus group discussions (FGDs) were held with 39 adolescents who were recruited independently of survey respondents. We aimed to recruit at least 30 adolescents for the FGDs as previous research has demonstrated four to six groups is sufficient enough to saturate key themes [32], while the team’s previous experience demonstrated that groups of four to six participants offer participants the most equitable opportunities to share ideas. The participants were divided into seven groups. Each group engaged in between four and six one-hour discussions via Zoom online video conferencing. The FGDs were each led by two or three trained facilitators who asked participants open-ended questions about their experiences during the pandemic (e.g. How are you coping? What are you doing to manage your time? How are you sleeping, eating, and exercising? What do you think about the current messaging from the government? How has remote learning affected you?). Discussions were audio recorded, transcribed (in five cases) and/or summarized through field notes entered into schema (all 30 cases), and analyzed (see Data Analysis) to capture participant experiences and reactions to the pandemic. Within a week of each FGD, participants were asked to complete the online survey (described above). Each group of FGD participants met at least four times between September 2020 until August 2021. Participants were offered a $15 Amazon gift card for each discussion they attended.

#### Art workshops

Study participants were given the opportunity to participate in online art workshops facilitated by local artists that were organized in partnership with the Art Gallery of Hamilton. The workshops were designed to offer a form of support to youth. That is, participants were encouraged to explore and share their feelings through art-based methods of expression. Data consisted of field notes, observations, and research team’s debriefings after workshops regarding participants’ apparent level of enjoyment and engagement. The discussions were not recorded or formally analyzed.

### Data analysis

Data analysis was based on a sequential exploratory design, wherein qualitative data was used to supplement and further explore relationships emerging from analyses of the mixed survey data.

Quantitative survey data pertaining to mental health and wellbeing, general time use and activity patterns (e.g. frequency of engagement in hobbies and games, frequency of deep social interactions, heaviness of workload, participation in study FGDs), physical activity, sleep, diet quality and food security, screen time use, and personal hygiene were analyzed in R (version 4.2.1). Survey response data on mental health and wellbeing, sleep, physical activity, leisure activities, diet quality, and screen time use were scored (See Supplementary file) and their component item scores were summed to develop a series of scales. The Cantril Ladders and the two scales used to measure mental distress (the CHU9D) and resilience (the SWEMWS) have previously been validated with young people in multiple populations [24–26], and these scales had high internal reliability in this sample (Cronbach's *α* = 0.83 and Cronbach's *α* = 0.85, respectively). The scales used to measure adolescent time use, health behaviours, and other activities have not previously been validated (with the exception of the diet quality scale), and generally had very low (in the case of the workload scale Cronbach's *α* = *0.18*) or middle-level internal reliability (Cronbach's *α* alphas ranging from 0.41 to 0.78; see Table 2 for the full list). Central tendencies and spreads of mental health, wellbeing, and health-related behavioural/activity scales were described and then compared across socio-demographic groups (e.g. age group, gender, high school versus secondary school attendance, neighbourhood) using the gtsummary R package (version 1.7.0) to identify overall trends and tendencies as well as possible causes or consequences of inequitable mental and behavioural health outcomes. Relationships among the health and wellbeing and health behavioural variables were also explored via a correlation matrix.

Qualitative FGD data were summarized and analyzed through 15–45 min debriefing sessions among the facilitators immediately following each one-hour discussion, in which main themes and ideas highlighted by the participants in each session were noted in a schema. Most notes focused on points related to our main research questions and to the questions in our interview guide, that is, how the pandemic was impacting adolescents’ mental health and wellbeing-related behaviours, and what could be done to support adolescent health and wellbeing through the remainder of the pandemic and beyond. Dominant, recurring, saturated themes appearing in the schema were discussed by multiple team members (JC, JM, DS, LM, SB, TW) over the course of three multi-hour analytical discussion sessions with all of these authors and several shorter meetings with sub-sets of these authors; relationships among themes were identified, and variations in theme contents and foci across discussion groups and through time were noted. Quotes illustrative of these themes were extracted from FGD transcripts or audio-recordings. Open-ended questions from the survey, which generated qualitative data, were also hand-combed by two team members (JC and LM) to assess the extent to which key themes from the FGDs reflected the views, priorities, perspectives, and experiences of the broader sample of respondents that completed the anonymous survey; these survey responses were also mined for quotes illustrative of the study’s main findings.

To identify global patterns associated with resilience across qualitative and quantitative data, we performed Factor Analysis of Mixed Data (FAMD; FactoMineR R package version 2.7 [33]). FAMD is a form of factor analysis (a common statistical method for simplifying our understanding of the variability in multiple, correlated variables of interest that “load” onto underlying unmeasured latent variables called factors). FAMD is a special class of factor analysis that allows for the bringing together of both continuous and categorical variables in the construction of factors. This method mathematically transformed our input variables into a set of linearly uncorrelated variables (factors), while maximizing the preservation of data variability. The first factor represents a new variable that explains the maximum variability in the data and the next factor explains the maximum variability orthogonal to the first factor. Data from each input variable of interest contributes a varying proportion to each of these factors. Finally, we made and visually inspected plots with these factors as the axes (factoextra version 1.0.7 [34] and ggplot2 version 3.4.0 R packages) and fit a linear model of our indicator of resilience and coping as a function of the variable loadings on the first factor (FA1).

## Results

### Sociodemographics

Summary statistics concerning key socio-demographic characteristics of survey respondents and FGD participants are presented in Table 1. There were 631 responses to the online survey, of which 565 were at least near-complete (had responded to questions on all pages/ sections of the survey except possibly excluding two open-ended, qualitative questions at the end), and 479 of these were from unique people who were eligible for study participation (adolescents, lived and/or attended school in Hamilton) and who were completing the survey for their first time.

**Table 1:** Socio-demographic descriptions of survey respondents and FGD participants

Socio-demographic Factor	Socio-demographic sub-group	FGD participants (*n* = 39)^a^	All survey respondents (*n* = up to 560, includes responses from respondents who did not fully complete the questionnaire)
**Mean age, years**		17.0	16.1
**Education, n (%)**	*secondary*	27 (69%)	458 (85%)
	*post-secondary or gap year*	12 (31%)	82 (15%)
**Pronouns:**	*she/her*	27 (69%)	300 (54%)
	*he/him or refused*	6 (15%)	164 (29%)
	*they/them or other non-binary*	2 (5%)	43 (8%)
	*missing*	4 (10%)	53 (9%)
**Racialization**	*white*	18 (46%)	384 (69%)
	*likely to experience racialization*	17 (44%)	170 (30%)
	*missing or refused*	4 (1%)	6 (1%)
**Neighbourhood poverty level**	*Low or middle range rates of poverty or lives outside city or missing*	37 (95%)	449 (82%)
	*Higher rates of poverty*	2 (5%)	97 (18%)

^a^Socio-demographic information as reported in surveys was missing from 10 out of 39 FGD respondents. Data on education level was available from field notes and FGD schema for all of these 10 participants. Data on pronouns and racialization and neighbourhood was filled in using field notes and FGD schema for 6 of them, but was missing/unavailable for the remaining 4. The FGD mean age estimate is based only on the 29 FGD participants for which we had corresponding survey responses

Seven FGD groups (four high school-aged; three post-secondary-aged) were formed, ranging in size from three to eight participants (total n for FGD participants = 39). Over 30 h of FGD audio recordings were captured and analyzed. These participants completed the survey and their open ended qualitative data responses were gathered and analyzed longitudinally, along with the FGDs. The median age of all (survey + FGD) study participants was 16 (ages ranging from 12 to 20). Although median age was the same, mean survey respondent age (16.1) was significantly (q^3^ = 0.026) younger than that of FG participants (17.0). This is likely a function of our sampling approach where we advertised the survey to all 13,000 high school students in the school district (which skews towards adolescents under 18), whereas we promoted the FGDs predominantly through social media, university message boards, and word of mouth (which likely skewed slightly towards university-aged adolescents). The racial composition of survey-only respondents and FGD participants differed significantly (q^3^ = 0.026): a greater proportion of survey respondents identified as white compared with FGD participants (69% vs. 46%), while more FGD participants identified as persons of colour compared with survey respondents (44% vs 30%). The neighbourhood poverty rate also differed significantly (q = 0.026), with FGD participants more likely to live in neighbourhoods with lower poverty levels compared to survey-only respondents (95% vs. 82%).

### Mental health, wellbeing and health behaviours

Overall/median scores and socio-demographic sub-group variations for mental health, wellbeing, and health behavioural indicators are reported in Table 2. Illustrative quotes related to each group of variables from FGDs and/or from the open-ended survey responses are reported in Table 3.

**Table 2:** Socio-demographic variations in mental health, wellbeing, and health behavioural indicators

	Summary		Pronouns					Educational Group				Racialization				Neighbourhood Poverty Rate				Chronbach's Alpha
**Characteristic**	Missing (%)	Overall	NB/pan^a^	He/Him^a^	She/Her^a^	p^b^	q^c^	Highschool^a^	Postsec^a^	p^b^	q^c^	Non-White^a^	White^a^	p^b^	q^c^	Low^a^	Med/High^a^	p^b^	q^c^	
**n**	0	560	43	149	300			458	82			176	384			225	280			
**Pre-pandemic Life Satisfaction**	9.8	7 (6, 8)	6 (5, 8)	7 (6, 8)	7 (6, 8)	0.016	**0.022**	7 (6, 8)	8 (7, 8)	0.076	0.10	7 (6, 8)	7 (6, 8)	>0.99	>0.99	7 (6, 8)	7 (6, 8)	0.94	0.94	
**Current Life Satisfaction**	9.6	5 (3, 6)	4 (3, 5)	5 (4, 7)	5 (3, 6)	0.001	**0.003**	5 (3, 6)	5 (4, 7)	0.065	0.10	5 (3, 6)	5 (3, 6)	0.55	0.77	5 (3, 6)	5 (3, 6)	0.45	0.68	
**Social Media Use**	17	3 (2, 4)	3 (2, 4)	3 (2, 3)	3 (2, 4)	0.031	**0.038**	3 (2, 4)	2 (2, 4)	0.48	0.53	3 (2, 4)	3 (2, 4)	0.52	0.77	3 (2, 4)	3 (2, 4)	0.33	0.68	
**Mental Distress**	7.7	21 (16, 27)	27 (23, 34)	19 (14, 25)	21 (16, 28)	<0.001	**<0.001**	21 (16, 28)	20 (15, 24)	0.061	0.10	21 (16, 27)	21 (16, 28)	0.85	0.94	21 (15, 28)	21 (16, 28)	0.51	0.68	0.8546
**Resilience**	10	21 (17, 24)	18 (16, 20)	23 (19, 26)	20 (17, 24)	<0.001	**<0.001**	20 (17, 24)	22 (18, 25)	0.069	0.10	21 (16, 24)	21 (17, 24)	0.56	0.77	21 (17, 24)	21 (17, 24)	0.55	0.68	0.8330
**Work Load**	21	3 (2, 3)	3 (1, 3)	3 (2, 3)	3 (2, 4)	0.002	**0.005**	3 (2, 3)	3 (3, 4)	<0.001	**<0.001**	3 (2, 3)	3 (2, 3)	0.66	0.81	3 (2, 3)	3 (2, 3)	0.20	0.56	0.1807
**Leisure Acitivties**	18	12 (8, 16)	13 (9, 16)	15 (11, 18)	11 (7, 15)	<0.001	**<0.001**	13 (9, 17)	8 (6, 13)	<0.001	**<0.001**	11 (7, 15)	13 (9, 17)	<0.001	**0.009**	13 (9, 17)	13 (8, 16)	0.50	0.68	0.5235
**Creative Engagement**	17	5 (3, 7)	8 (5, 9)	4 (2, 6)	5 (4, 7)	<0.001	**<0.001**	5 (3, 7)	5 (2, 6)	0.064	0.10	5 (3, 6)	5 (3, 7)	0.47	0.77	5 (3, 7)	5 (3, 7)	0.64	0.70	0.4111
**Sleep Quality**	17	18 (13, 22)	19 (16, 23)	16 (11, 21)	18 (14, 22)	0.008	**0.013**	18 (12, 21)	18 (15, 21)	0.21	0.25	18 (14, 22)	17 (13, 21)	0.19	0.67	17 (13, 20)	18 (13, 22)	0.034	0.13	0.7785
**Physical Activity**	18	10 (7, 14)	10 (5, 12)	10 (8, 15)	11 (7, 14)	0.28	0.31	11 (7, 15)	9 (7, 13)	0.027	0.10	9 (7, 13)	11 (7, 15)	0.037	0.20	11 (8, 15)	10 (7, 14)	0.003	**0.016**	0.6850
**Diet Quality**	13	26 (20, 30)	25 (16, 32)	25 (21, 30)	26 (20, 30)	0.66	0.66	26 (20, 30)	25 (21, 29)	0.60	0.60	26 (21, 31)	25 (20, 30)	0.25	0.67	27 (22, 31)	24 (18, 29)	0.001	**0.013**	0.6515

^a^n; Median (IQR)

^b^Kruskal-Wallis rank sum test

^c^False discovery rate correction for multiple testing

**Table 3:** Illustrative quotations demonstrating adolescent’s experiences throughout the COVID-19 pandemic

**Quotations about participants’ engagement in leisure activities**
“I am glad I have hobbies like drawing, which can be done despite the lockdown” “A pond by my house recently froze over and so I just… began going down and skating on it a few times this week. It's… an escape and a way to…. numb my mind” “I’ve been playing minecraft a lot on the school iPad with my friends, which gives us all an opportunity to connect” “I now value my local parks where I played soccer with others… I now ride [local bike share] bikes more often” “Since it is currently between first and second semester, the students got the week off. I've taken the time to relax and do self care activities (cleaning my room, bake and cook for my family, do at home workouts, and help out my family with more chores since I have more free time)”
**Quotations about participants’ reduced physical activity**
“I dance but because gyms and different places aren’t open,[…] it’s been very hard to do that. I have my tiny bedroom and that’s about it for space, so exercising hasn’t really been a thing.” “Now that school and hockey are still not really happening, I’ve lost all motivation and can’t bring myself to do exercise.” “I used to like going to the gym after class… and now it’s basically impossible. I can’t really set up a home gym right because it’d be almost impossible to get my own equipment and stuff. So that would obviously [be a] detriment [to] my health ‘cause I’m not really able to exercise anymore”
**Quotations about participants’ dietary habits**
“I used to eat more vegetables – a more balanced diet. I’ve been leaning towards things that make me feel good in that moment.” “It has been very inconsistent, where some days I would eat so much and some days I would go the whole day without eating anything.” “It’s a lot easier to fall into temptation and fall into, like, junk food… when you’re in isolation”
**Quotations about difficulties with online schooling**
“Well my marks dropped substantially. I’ve been an honours student for three years in a row, and suddenly I’m getting 60 s that out of nowhere dropped to 40 s… I’ve had multiple mental breakdowns about school.” “Some work is too hard to understand without the one-on-one with a teacher. And, half the time the programs or internet doesn't work. I used to like going to school.” “I’d rather be in school to learn because it’s hard to listen and stay away from the Xbox when I have to login to class. Doing school work on the [tablet] is hard to do. I have a hard time filling in and sending assignments. When someone isn’t right there to help me with schoolwork, I lose interest and just don’t want to do it.” “I have noticed that because of the lockdown and school being online, I have experienced more stress than I am used to. Because I do school in my bedroom, I can never feel fully relaxed because my safe space has also become my work place. This causes a lot of stress and fatigue and I find it hard to understand the emotions that I am feeling.”
**Quotations about greater adult-level responsibilities, disappointment about lost milestones, and feelings of loneliness, isolation and “burn-out”**
“I feel so unhappy and cheated to lose the experiences I so desperately long for: dances, assemblies, spirit weeks, club meetings, etc. I wonder whether I'll ever get those experiences that you remember for the rest of your life, even the bad prom dresses and horrible dance songs.” “Well I have 0 friends. I went to a new high school and not allowed to do much. Which has really had an impact on me. I also have a busy family. So if you have any idea on what it’s like feeling unimportant and not wanting to tell people and bombard them. It’s me.” “As a high school senior I am missing out on everything I ever looked forward to in high school and I feel our parents and teachers don’t validate our feelings on that… And without the output of sport and seeing friends my mental health and motivation to do anything are horrible.” *“*I just became a hermit, and like, only go to work and do school”
**Quotations demonstrating adolescent resilience throughout the pandemic**
“Lockdown has left a lot of time for self reflection, so I've discovered new career paths and the feeling of loneliness and loss during lockdown… I couldn't see my grandpa for the last time and that made me realize a lot about society and what my family means to me, it has become more important to cherish what's there.” “At the beginning of lockdown, it was horrible. I found myself binge eating to cope and was super depressed that I wasn’t able to go out and see my friends and live a “normal life”. Shortly after, I (and my family) realized that we needed to take control of ourselves, so we decided to lose weight to better our lives… I’m happier with my body, I feel healthy, and it is so much better for my mental health not being in all the gossip and stupid stuff at school. I do want it to go back to normal but I really think lockdown has been good to me.”

Broadly, the overall health of the survey respondents (the majority of whom responded during a time period of strict public health restrictions and major disruptions to social, educational, and other features of everyday life) and of the participants who engaged in FGDs during this same period (December 2020 to March 2021) was poor. Most respondents and participants reported lower current life satisfaction than pre-pandemic life satisfaction, and many reported high levels of mental distress. Furthermore, the majority reported inadequate sleep, inadequate physical activity, excessive social media use, and some struggles around eating well. Below, we flesh out these points about health behaviours in the sample as a whole, highlighting any striking inequities pertaining to a given theme or behavioural domain. We then report on gender-based inequities in our indicators of mental health and health behaviours. We pay extra attention to gender because gender sub-group appears to be among the most consistent predictors of variation across a wide range of the mental and behavioural health indicators we measured.

27% of survey respondents reported sleeping less than 8 h/night, thereby not meeting minimum recommendations for healthy adolescents [35]. 86% of survey respondents reported increased recreational screen time relative to pre-pandemic levels, where 55% of survey respondents reported using one or more major social media platforms at least once an hour. Only 4% reported never using social media platforms. Social media use frequency was generally similar between high school (n = 458) and post-secondary respondents (n = 82), with the modal response being 2 (I use/check social media a few times a day) and the mean response being somewhere between 2 (I use/check social media a few times a day) and 3 (I use/check social media at least once an hour) for each group. Notably, respondents with the highest frequencies of social media use scored higher mental and emotional distress scores—an average of 10 points higher on a 40-point scale—than those with the lowest frequencies of social media use. These effects are statistically independent of respondent’s age, gender, post-secondary attendance, and racialization.

Most (> 99%) survey respondents engaged in some form of leisure activities, although two (< 1% of sample) had scores of zero on the leisure activities scale. A higher score on the leisure activities scale indicates more engagement in activities like playing sports; dancing and/or doing martial arts; recreationally running, walking, hiking, and/or cycling; participating in online and/or offline gaming and puzzle-solving; and/or doing handiwork or fine arts. Respondents from high school were significantly (q^3^ < 0.001) more likely than post-secondary students to have higher scores on the leisure activities scale. White respondents were also significantly (q^3^ = 0.008) more likely to report having participated in more leisure activities than non-white respondents. Quotations about participants’ engagement in leisure activities can be found in Table 3.

83% of survey respondents and most FGD participants reported below guideline levels of physical activity for Canadian youth (i.e. 60 min/ day of “moderate to vigorous physical activity”) [36] and 39% of survey respondents reported less than 15 min/day of any form of physical activity. Adolescents self-reported that their activity levels were low pre-pandemic and when asked if they moved their bodies more, less, or the same as pre-pandemic, most survey respondents were likely to say less than more. Notably, physical activity patterns varied among survey respondents socio-demographically; physical activity scores were lower among respondents from neighbourhoods with lower poverty rates compared to those from neighbourhoods with higher poverty rates (q^3^ = 0.015). FGD participants reported imposed restrictions as reasons for disruptions to their normal physical activities (e.g. no longer being able to go to the gym or play organized sports due to public health-mandated closures). Some also noted that lack of structure to their school/work days disrupted many of the positive physical activity habits they had in place pre-pandemic. Some quotations reflecting participants’ physical activity habits can be found in Table 3. Many of the participants suggested lack of physical activity was a driver of poor sleep, reduced interest in healthy food, poor body image perception, and poorer-than-usual mental health and worse-than-usual life satisfaction.

Generally, survey respondents’ diets met or were near Health Canada recommendations for whole grains, fruits and vegetables, and sources of lean animal protein intake. Few respondents reported eating pulses regularly. Most respondents reported eating some form of junk food every day or almost every day. In the FGDs, some participants noted that they were generally eating more family meals or meals with roommates and eating out less as a result of pandemic-related public health restrictions. Some had also set specific goals for themselves to learn to cook or to become better cooks, and developed new structures and routines around preparing nutritious food. Some FGD participants reported stress eating, eating out of boredom, forgetting to eat, and developing worse relationships with their bodies and suffering negative body images. At least five of these participants appeared to have developed new or worsening eating disorders and at least one disclosed having developed a possible alcohol dependency. Some example vignettes of these experiences, as described by the participants, can be found in Table 3. Much of this variation and complexity in adolescent diet quality is related to socio-demographic variation. Survey respondents from neighbourhoods with higher poverty rates had significantly lower overall diet quality than respondents from neighbourhoods with lower poverty rates (q^3^ = 0.012). Similarly, respondents from food insecure households had significantly lower overall diet quality scores than respondents from food secure households (q^3^ = 0.010).

Some of the longest school closures in Canada occurred in the province of Ontario [37]. Elementary and high school students lost approximately 28 weeks of in-person learning between the declaration of the pandemic and May 2022 [38]. The majority of FGD participants found the online school environment psychologically challenging. One survey respondent said *“All [online schooling] has done is added more stress to my everyday life and makes me dread getting up in the morning… I used to excel in my classes… now I don’t care as long as I pass my classes.”* Adolescents expressed frustration at the lack of supports available to keep up with, and to understand, online course materials. Linked to this was a lack of structure and routine that in-person school provides. Many students reported feeling disengaged or having “checked out” of school. The cumulative impact of less sleep, less physical activity, increased screen time (including social media use), lack of clear markers of progress and time passage, social isolation and increased overall stress led to burnout for some adolescents. In this vein, some FGD participants expressed that they had reached a limit in their ability to cope. Quotations where participants discussed their experiences with online schooling environments can be found in Table 3.

In addition to the changes in health and wellbeing reported quantitatively in the survey, the FGDs revealed additional challenges faced by youth, including dealing with more adult-level responsibilities, and feelings of loneliness, isolation, and “burn-out”. Participants frequently reported having missed out on key social and cultural milestones. While some events (e.g. driver’s license, first job) could take place in the future, other events (e.g. graduations, milestone birthdays, entering first year of university) had passed and could not be experienced again. Some FGD participants reported being able to self-organize small, physically-distanced graduation events, but many other adolescents lacked the relational, financial, or psychological resources to do this. Participants told us that they experienced increased difficulty with or changes in relationships with adult decision makers (i.e. parents, teachers, bosses, and higher-level policymakers). This was often expressed as a lack of support (e.g. bosses unwilling to support adolescent employees who sought to enforce masking and vaccination rules) and accountability (e.g. no consequences for adult leaders who failed to lead effective strategies to account for the loss of experiences or learning). Participants reported that they were made to shoulder adult-level responsibilities in the workplace, as many of them held positions as essential, frontline workers (e.g. enforcing mask mandates in grocery stores) without commensurate power/authority, life experience, or compensation. Quotations about these challenges can be found in Table 3.

There were several significant differences reported between individuals identifying as he/him (“boys”), and those identifying as she/her (“girls”), and/or non-binary (“NB adolescents”) across most variables measured (see Table 2). With respect to mental health and wellbeing, boys expressed the highest levels of both pre-pandemic (q = 0.022) and current life satisfaction (q = 0.003), followed by girls and then followed by NB adolescents. Boys also had the highest resilience scores (q^3^ < 0.001), girls had middling resilience scores, and NB adolescents expressed the lowest level of resilience at the time of sampling. Additionally, boys reported significantly lower levels of mental distress than girls, whereas NB adolescents reported the highest levels of mental distress (q^3^ = 0.001). Qualitatively, however, in both FGDs and survey responses, boys who reported poor mental health appeared more extreme in their presentations of distress and more likely to report engaging in negative coping strategies (e.g. more extreme language used when describing negative emotions and painful narratives; possible substance abuse in two cases).

With respect to health behaviours and time use, boys were significantly (q^3^ < 0.001) more likely to report participation in leisure activities, whereas both girls and NB adolescents were significantly (q^3^ < 0.001) more likely to report having participated in creative activities. Sleep quality and workload also differed by gender, with both girls and NB adolescents reporting significantly (q^3^ = 0.011) worse sleep than boys and significantly more work than boys (q^3^ = 0.005). NB adolescents appeared unique in their reported social media use, which was significantly higher than that of both boys and girls (q^3^ = 0.038). There were no statistical differences among gender groups in diet quality or physical activity.

### Resilience

Comparing overall life satisfaction pre-pandemic with that during the pandemic, 7% of survey respondents reported no changes to their wellbeing and 13% reported *higher* overall life satisfaction scores during the pandemic compared to how they recalled feeling pre-pandemic. Therefore, one out of five survey respondents reported being just as, or more, satisfied with their lives during the pandemic. Consistent with this, some FGD participants reported being able to cope with their challenges in a resilient and positive way. They identified school closures and restrictions on social interactions as an opportunity to get to know themselves better and/or to redefine and deepen their relationships with others. Some said they learned new skills or experimented with new hobbies. One participant described the summer of 2021 as an opportunity to *“completely renovate [her] whole identity.”* Other quotations related to resilience and positive coping are reported in Table 3.

**Fig. 1:**
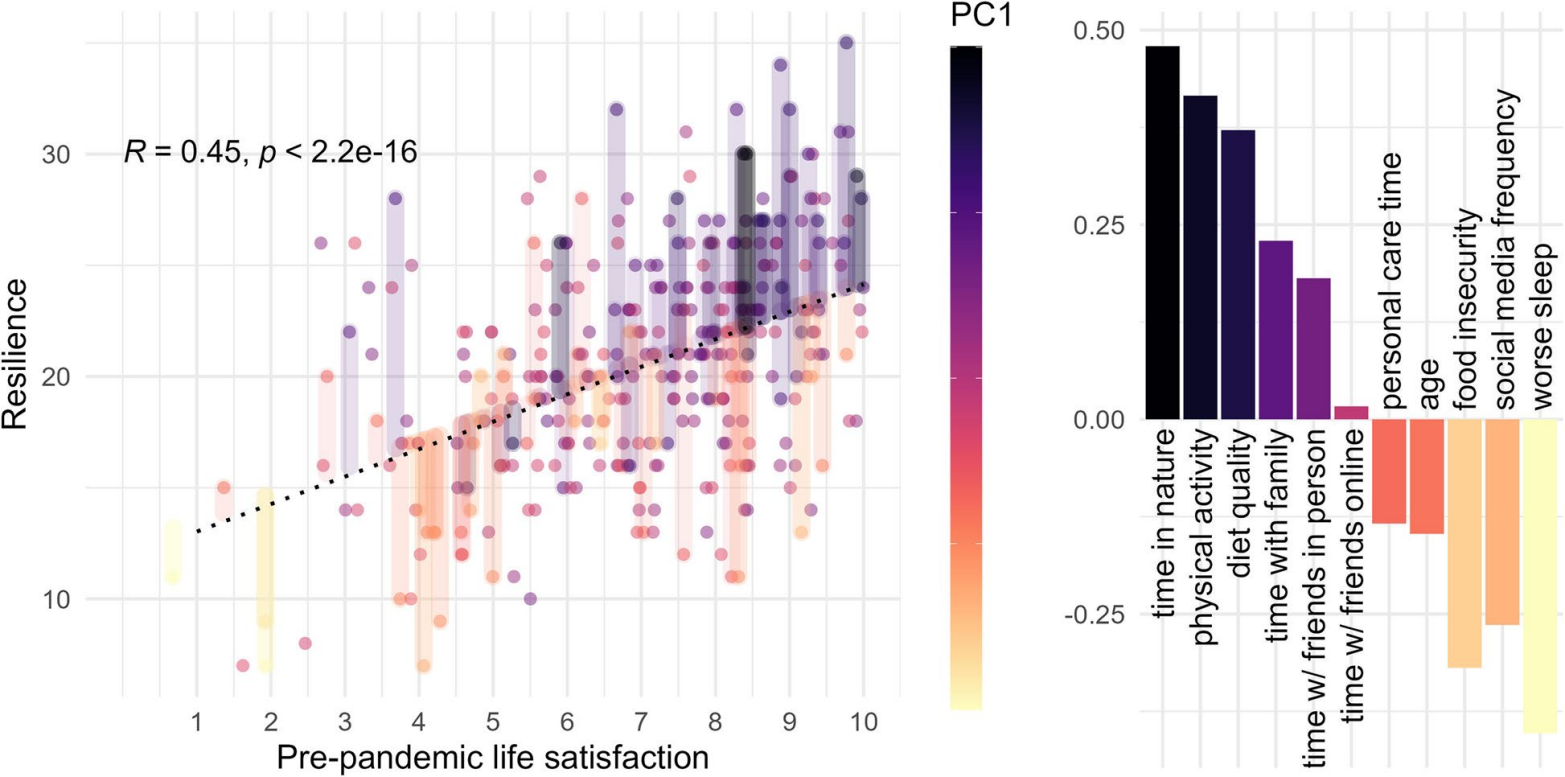
Behavioural and socio-demographic factors modulate relationships between pre-pandemic life satisfaction and resilience during the pandemic. Recalled pre-pandemic overall life satisfaction is positively correlated with current overall individual resilience (derived from the SWEMWS) across all individuals (R = 0.44, p < 2.2e-16, n = 328). Significance was assessed by a linear model. Factor Analysis of Mixed Data (FAMD) dimensionality reduction of behavioural and socio-demographic factors was performed and their relative contributions to Factor 1 (FA1) are shown (right). [Capacity for resilience] FA1 values of each individual are indicated by a continuous colour scale, and residuals are shown for individuals > 1 standard deviation from the mean of FA1 (left). The mental resilience score of individuals with high or low [capacity for resilience] FA1 values tends to be respectively greater or lesser than predicted by recalled pre-pandemic life satisfaction as shown by black and purple residuals above the line of best fit and orange and yellow residuals below the line of best fit. This indicates that factors with positive contributions to [capacity for resilience] FA1 (e.g. more time in nature, more physical activity, more time with family and friends in person) are beneficial to mental resilience while factors with negative contributes to [capacity for resilience] FA1 (e.g. increasing food insecurity, increasing social media frequency, worse sleep) are detrimental

To understand why some adolescents had positive perspectives during this crisis, we used the first factor (FA1) derived from the Factor Analysis of Mixed Data (FAMD). The first factor in the FAMD analysis [FA1] is a composite measure of socio-demographic and health-behavioural factors expected to underpin variation in resilience in the face of crisis. We termed this factor *capacity for resilience.* This accounted for 16% of variation among these variables. We modeled our indicator of overall resilience as a function of pre-pandemic life satisfaction and capacity for resilience [FA1] (Fig. 1).

While multiple factors were associated with variation in resilience in our participants, the strongest positive associations were with spending more time in nature and being more physically active. The strongest negative associations were with poor/inadequate sleep, followed closely by high frequencies of reported social media use and food insecurity. Consistent with this, FGD participants indicated that increased screen time was generally impacting their sleep, physical activity, and sense of motivation to do work and to engage socially; but, they did not specifically identify frequency of social media use as a determinant of their mental health or health behaviours. Several FGD participants reported taking walks in nature as a way of de-stressing, especially when other outdoor activities (e.g. sports) were prohibited. The participants that engaged in our online art workshops described these activities as providing relief from their daily stresses. The workshops provided an opportunity for participants to express themselves creatively. Participants reported that the workshops offered new outlets for self-expression. Overall, participants reported that their experiences with the FGDs were overwhelmingly positive, with many indicating that they valued having a forum to compare experiences with their friends and peers in a safe and informal environment, and that they felt prompted to have deeper discussions than might be the case in an unfacilitated setting.

### Longitudinal observations

**Fig. 2:**
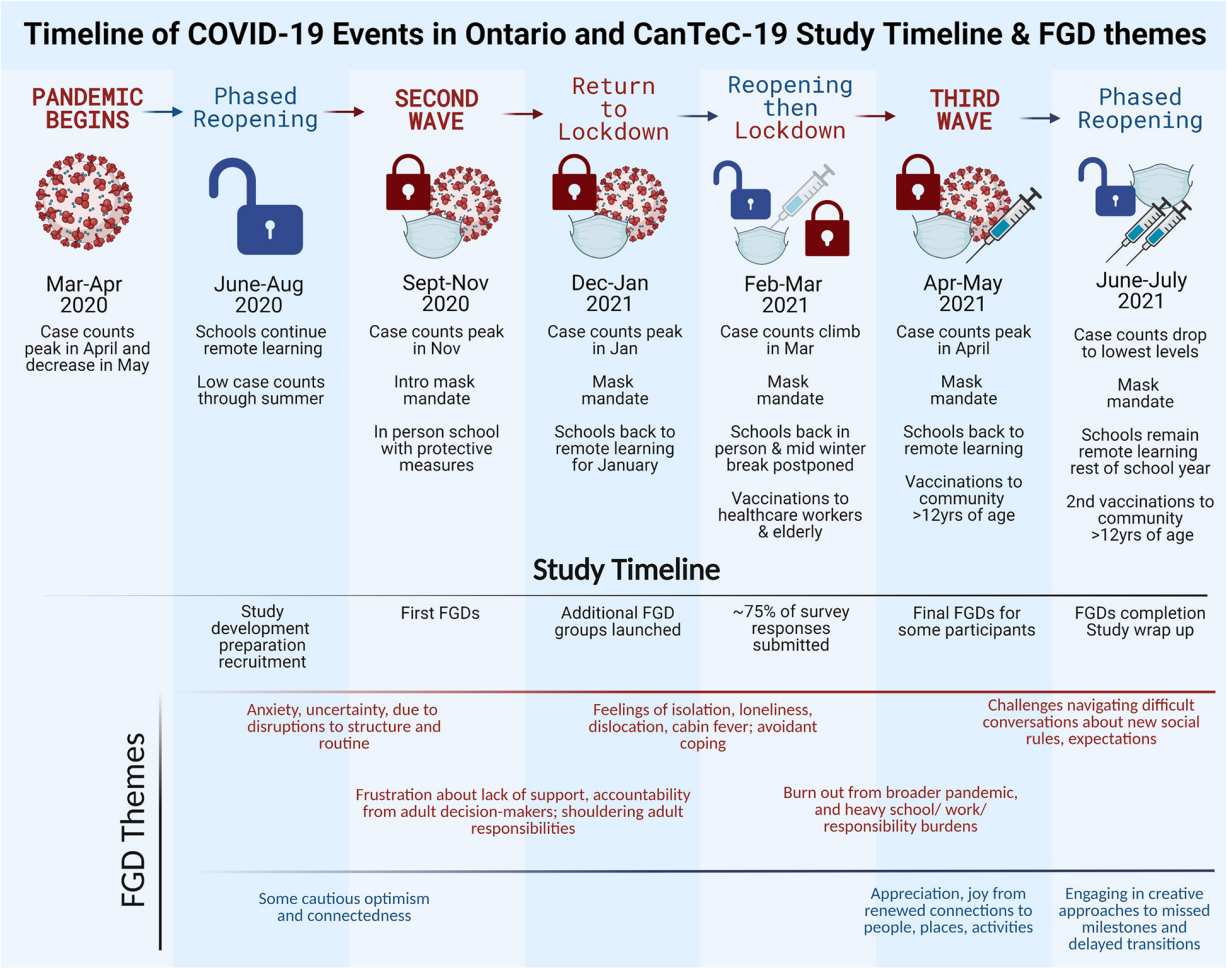
Key COVID-19 events in Ontario, Canada linked to study activities and themes from CanTeC-19’s FGDs. Spanning prior to (March to August 2020) and through the study period (September 2020 to August 2021), this timeline gives a fulsome description of the constraints imposed on adolescents during the study period. By linking COVID-19 events with the dominant themes from CanTeC-19’s 28 FGDs, it is evident that imposed restrictions were associated with adolescents’ attitudes and perspectives. Generally, themes surrounding negative attitudes and challenges for adolescents (outlined in red) are connected to periods of lockdown, whereas themes associated with optimism and resilience (outlined in blue) are connected to periods of phased reopening

One particular strength of our study was that we met participants in the FDGs repeatedly during the study period (over a 7–11 month span, depending on the group). Thus, we were able to observe changes in adolescent attitudes and perspectives through different waves of the pandemic (Fig. 2).

FGDs and open-ended survey responses revealed that periods of increased restrictions/lockdowns were associated with greater reports of mental distress. We heard about profound feelings of isolation, dislocation, and loneliness; reports of “cabin fever” and feelings of being “trapped”; and a desire for freedom when restrictions were tight. Many expressed frustration, stress, and anxiety about changes to schooling structure. Some reported anxiety about illness, about responsibility for vulnerable family members and friends, and for being given too much responsibility at their “essential” jobs (e.g. for managing non-compliance to pandemic/masking rules). In the participants’ own words: “*The lockdown has almost ruined my life, feels like prison. I lost a lot of friends, and feel like I’m in a never-ending depression*”; “*It sucks that I’m probably losing the best years of my life and I’ll never get them back. I’m miserable.*”; and, from someone who noted that they dropped out of grade 12 during the 2020–21 academic year “*Cancellation of in-class school work is the reason I left [secondary] school*”. In contrast, by late spring 2021 when restrictions were loosened significantly and as vaccination against COVID-19 became widely available to the general adolescent population in Canada, attitudes expressed in FGDs were perceptibly more positive and optimistic, and many participants had begun to develop their own solutions to many obstacles. For example, some participants began engaging in difficult conversations about behaviours and expectations with their friends and family, or planned harm-reducing ways to mark milestones like birthdays and graduations with key parts of their social support networks. However, as vaccination rates climbed and public health restrictions relaxed in April-June 2021, conversations with friends, family, and co-workers became more complex. Participants reported difficulties navigating how to interact with others within their social networks in stressful situations (e.g. discussing vaccination status, comfort with wearing masks, physical distancing, etc.). Taking place during a critical period for adolescent development that can already be socially awkward, some adolescents appeared to lack the tools to navigate these difficult conversations. Connections between the different waves of the pandemic and FGD themes are also shown in Fig. 2.

## Discussion

The COVID-19 pandemic has altered the environmental conditions shaping adolescents’ developmental markers. For the majority of adolescents, these new conditions were and continue to be relatively challenging. We found that Canadian adolescents (aged 12–20) in our urban/ peri-urban sample faced numerous barriers to good mental and physical health during the pandemic. Consistent with other reports [16, 39], we observed that the closure of schools, halt in extracurricular activities, lack of routine, and increased screen time were associated with worse health behaviours and poorer mental health scores.

Our data suggest that adolescents got less sleep, had fewer opportunities to have meaningful conversations about health and wellbeing, and used screens and social media more than would be the case in an optimally-healthy and well-supported adolescent population [40]. Furthermore, the majority of our participants were active for far less time than recommendations for healthy Canadian children and youth [36]. These poor health behaviours were likely connected with one another. Many of our FGD participants noted that loneliness and lack of in-person social engagement led to increased late night social media and general screen-time use, which led to sleep disruptions, which led to lack of energy and motivation to exercise and to do school work, which led to poor wellbeing, which led to more loneliness and late night social media use and sleep disruptions – a vicious cycle. Previous research in adolescent sleep and physical activity during the COVID-19 pandemic has also hypothesized similar connections [41, 42].

Our study suggests that the pandemic had a variable effect on adolescent diets. Our survey data suggests that participant diets were similar to Health Canada’s recommendations for whole grains, fruits and vegetables, and lean animal protein intake. However, adolescents who experienced food insecurity and/or who lived in areas that were more impoverished had poorer diet qualities. FGD participants reported that the pandemic affected their eating habits in various ways. Some discussed eating out less and becoming more interested in improving their cooking skills, while others discussed poor eating habits, such as forgetting to eat meals, or eating due to stress or boredom. This is consistent with the mixed or multi-directional findings of other investigations of pandemic-related changes in adolescents’ eating behaviours [43].

Our study suggests that the pandemic has had a heterogeneous impact on adolescents’ mental health. Consistent with other reports [15, 41, 42], some adolescents experienced more loneliness, stress, and anxiety due to greater adult-level responsibilities, loss of milestones, isolation, burnout, and changes in their relationships. This was especially pronounced during times of increased restrictions and lockdowns. In contrast, some participants experienced improved quality of life and/or health behaviours during the pandemic. Interestingly, about 20% of survey respondents reported no change in or improvements to their wellbeing associated with the pandemic. Participants identified multiple contributors to their resilience and strength, which included a break from the usual bombardment of social interaction and stimuli characteristic of in-person high school and post-secondary life. In this regard, adolescents were granted more time to relax, and were given relief from school phobias or bullying [15, 44]. This afforded some adolescents the opportunity to reflect on their lives, their relationships with family/friends, and their priorities. Being housebound also appeared to support new and deeper connections between the participants and others in their households, often accompanied by a new sense of caregiving and domestic responsibility. Growing numbers of investigations on the COVID-19 pandemic’s impact on adolescent health and health behaviours have urged researchers to investigate the complex factors associated with increased adolescent resilience during the pandemic [45]. Candidate contributors to resilience include adolescents’ socioeconomic status; gender; barriers to being healthy prior to the pandemic (i.e. pre-existing mental or physical conditions, lack of support); and healthy relationships with peers and family [44, 46, 47]. Our analyses show that adolescents who connected in-person with friends and family, went out in nature, were regularly active, used social media less, and managed to get sufficient sleep were more likely to report higher-than-expected scores on a reliable indicator of wellbeing. We interpreted this as being indicative of relatively high resilience. Likewise, respondents with poorer/inadequate sleep, high frequencies of social media use, and food insecurity were interpreted to be less resilient or less well-equipped at engaging in healthy coping.

During adolescence, the frontal lobe entrenches skills and lifestyle habits that can shape future behaviours [6]. As such, forming positive habits early in life may reinforce engagement in behaviours that improve health and wellbeing in adulthood [48]. Indeed, it is this preconceptional period that has been proposed to set the stage for health behaviours that impact future generations [49]. Isolation due to school closures and social “lockdowns'' prevented adolescents from acquiring or practising many face-to-face social, emotional, and cognitive skills, and other life skills, including the ability to structure a daily routine [50–52]. The effects of COVID-related school closures and other restrictive measures may have been further exacerbated in adolescents who have disabilities or a limited access to resources [16].

With all of the foregoing in mind, we developed a series of recommendations (Table 4) that can be implemented globally during crises, including global pandemics, to ensure adolescents have a good foundation for developing and/or maintaining beneficial health behaviours. Specifically, we urge those involved in supporting adolescents to accommodate the sleep, nature, social, activity, and mental health needs of adolescents. For example, schools and policymakers should consider later class start times, regular breaks for physical activity (e.g. “recess break” in high school), more social opportunities during the day/evening (e.g. organized meet and greets), outdoor classes (increasing adolescents’ time in nature and physical activity to build resilience), and assistance with independent learning (i.e. extra help sessions for classes and university applications, and flexible due dates). Furthermore, the global increase in youth mental health challenges reveals that adolescents need a solid foundational support system during times of crisis; our FGD participants were overwhelmingly positive about how the focus groups provided support for them. Peer support groups may be a promising intervention strategy to incorporate into school settings, extracurricular activities, or clubs. Participants who engaged in our FGDs suggested that having a semi-structured and facilitated forum to discuss their lives and health with their peers (i.e. focus groups, art workshops) was helpful in building motivation and avoiding rumination. This is consistent with previous reports that suggest that peer support groups have beneficial effects on adolescent mental health during crises [53, 54]. It is also worth considering the benefits of completing art-based activities during peer support groups. Participants who engaged in our art workshops suggested that they offered new opportunities and methods to express themselves. Art stimulates areas of the brain associated with stress and mood regulation [55], and has been found to facilitate social cohesion and inclusion [56], which can strengthen relationships within a community.

**Table 4:** Recommendations to improve adolescents’ wellbeing during crises

**Public Health Support Recommendations**
•** Greater focus on the capability of youth**—The adolescents we spoke to in our focus groups were following public health rules, even at the expense of their mental and physical well-being. In recognition of this, public health messaging should place greater focus on the capability of youth to take positive, constructive actions •** Messaging that is more accessible**—Adolescents told us they would like public health messaging, including information about vaccines, to be more accessible. Since most adolescents use news websites and social media to access pandemic-related information (rather than public health or other government sources), to reach youth audiences, public health needs to provide health-related information in a centralized place on youth-favoured platforms using credible voices. This would also address concerns we heard, around who or what to trust. Furthermore, using infographics and age appropriate language would make messaging easier to understand •** Engaging youth and rebuilding their trust in public health**- Some adolescents became disengaged from COVID-19 news and public health messaging as the pandemic continued. They found the messaging overwhelming and confusing instead of helpful. Work needs to be done to engage youth and rebuild their trust in public health and other government institutions. Youth ambassadors, or better yet, successful influencers and content creators with direct connections to public health could be used to engage youth
**School Support Recommendations**
•** Increased opportunities for participation in decision-making bodies**—Our focus group interactions revealed that we had engaged a thoughtful and aware group of young people. They were eager to share their thoughts and feelings and had actionable suggestions about how their education could be improved. Involving adolescents in decisions that affect them (e.g. academic structure, school safety) could lead to more positive outcomes for adolescent health and wellbeing. Ensure that youth, including those who would not necessarily self-nominate for leadership positions, are able to participate on boards, school committees or other decision-making bodies. Using an age-appropriate way of communicating with youth would be most successful (e.g. through relevant social media) •** Improving social opportunities**—Educational governing bodies should consider how to “make up” for the loss of important social opportunities. Rather than prohibiting large-scale social events, safe, supported and sanctioned group activities would allow youth an opportunity to make up for missed socialization that has occurred during the pandemic • Improving (virtual and in person) classroom conditions—For in-person learning environments, students were concerned about the health impact of large class sizes and schools being in poor condition. Investments in reducing class sizes and improving classroom conditions (particularly regarding ventilation and air filtration) would provide long-term savings for public health. For online learning environments, educators need to ground-truth which online models work, and which don't work, for which students and under which circumstances; flexible, self-paced, online learning environments work well for only a small subset of students •** Increased Support for Learning and Accommodating Adolescents' Sleep, Nature, Social, and Activity Needs**—Students told us they needed more support. Specifically, students mentioned: needing assistance with independent learning (e.g. providing an outline for semester plans), wanting extra help sessions for classes and university applications, and wanting flexible due dates. Schools could also consider accommodating the sleep, nature, social, and activity needs of adolescents. For example, they may consider varying class start times (e.g. later for high school), building in regular breaks for physical activity (e.g. recess in high school), creating more social opportunities during the day/evening (e.g. organized meet and greet), and having outdoor classes (e.g. nature/physical activity) to build resilience •** Developing peer support groups**—Our focus groups were reportedly a huge help to participants, providing them with a space that was supportive and deeply cathartic. Furthermore, guided group discussions with friends may help address heightened anxiety when students transition from elementary to high school and high school to post-secondary. Educators could consider designing and offering similar peer-to-peer and participatory support groups as a way to support students who are struggling or during times of uncertainty (i.e. during transitions or other crises). Adding an art-based element to them may also be beneficial; the art-based workshops we offered during our study were overwhelmingly positive experiences for adolescents who participated. These workshops can provide a much-needed outlet for creative self-expression and help adolescents deal with challenges in a different way
**Community Support Recommendations**
•** Advocates at work**—Some adolescents gained confidence by shouldering adult-level responsibilities in the workplace. However, many need advocates at work to help deal with the increased risks of enforcing ever-changing public health rules • Providing opportunities for work placements and connecting with community—Individuals that worked and/or deepened their social connections fared better than their peers who did not. These opportunities help adolescents find meaning and a sense of purpose. This being said, finding job opportunities and maintaining connections have been difficult for many youth. As such, youth could benefit from increased use of co-op work placements, access to employment-focussed councillors through schools and post-secondary institutions, and community-based social clubs or adolescent-focused activities organized within the community could be used to facilitate connections •** Improved accessibility to mental health supports**—Adolescents showed various levels of mental health impacts because of the pandemic, but some may not have sought help. Availability and accessibility of mental health support for adolescents’ needs to be communicated more broadly. The supports need to be made accessible for those who find it challenging to access. Easy access for students to counselors or social workers in schools and post-secondary institutions would provide more direct and immediate help • Promotion and support of community-based nature engagement—To promote and support family or community-based nature engagement, advertise and encourage the use of local conservation areas or hiking areas by making passes free to borrow from libraries, schools, or community centres • Access to pets and animal care—Looking after a pet provided some comfort to adolescents during the pandemic. Not everyone is able to have a pet, however, access to pet and animal care could be provided more broadly

### Study limitations

Our study is not without limitations. We used convenience samples (both for the survey and the FGDs) to make tentative inferences about the effect of the pandemic on Canadian adolescents. Furthermore, for respondents under 18, only individuals whose parents were able to understand a consent form written at a Grade 8 level of English were eligible to participate. We therefore likely excluded potential participants/respondents who were from non-English-speaking newcomer families, and possibly under-representing an already-marginalized portion of the population. We note, though, that despite this language-related limitation, both the FGDs and the survey represented or even over-represented sub-groups of adolescents expected to be under-represented in health research (especially adolescents of colour). In addition to this, because we engaged in FGDs on Zoom and asked participants to complete an online survey, our sample likely excluded individuals who had limited access to the internet. Despite this, our findings are consistent with those in other studies using representative samples [15, 16, 39, 41–43]. Given that the consent/assent forms, the survey, and the FGDs were offered only in English and the participants in this study were all living and/or attending school in the city of Hamilton, the results are likely not generalizable to the national Canadian adolescent population. Hamilton, however, is a socio-demographically, statistically average Canadian city [19], and thus this single-population study might be more generalizable than other single-population Canadian studies. Lastly, our small sample of FGD participants somewhat over-represents the perspectives of older, post-secondary-aged adolescents, adolescents with she/her pronouns, and non-white adolescents relative to survey respondents. Nevertheless, the data collected amongst the FGD participants is broadly and thematically consistent with the data we collected through the survey, suggesting that many of the key ideas articulated by the FGD respondents reflect some of the experiences and perspectives of a wider group of adolescents living and/or attending school in Hamilton, Ontario.

### Strengths

Our study has highlighted the importance of qualitative and discursive studies in supporting survey-based quantitative data in investigations of the impact of a significant life event on a group of people. While a growing body of literature investigating COVID-19 impacts on adolescent health framed this study, exploring the effects of the pandemic without a priori hypotheses enabled us to look beyond assumptions and consequently revealed that many adolescents were given the opportunity to develop resilience and explore factors associated with resilience. As such, we urge researchers to recognize the importance of observational studies and use these tools to understand the impacts of ongoing and future crises (e.g. the 2022 Russian invasion of Ukraine, the climate crisis).

The combination of longitudinal and cross-sectional data to understand how the pandemic has affected adolescents is another study strength. Using focus group data, we explored the impact of public health measures on adolescents as they were implemented and later rescinded. With a relatively large sample of cross-sectional survey data, we explored how adolescents from different demographics fared at specific time points throughout the pandemic. Finally, combining qualitative and quantitative data together allowed us to explore topics that were highlighted in surveys while the survey allowed us to ensure that the data that we collected from the FGDs was to some extent representative of Hamilton's larger adolescent population.

## Conclusions

Our analyses suggest that, in response to the COVID-19 pandemic, adolescents faced significant challenges to maintaining their mental and physical health. The majority of adolescents engaged in far less than recommended levels of physical activity, had poorer sleep, and engaged in more screen and social media use. However, some adolescents demonstrated better outcomes than others. Our study suggests that adolescents who connected in-person with friends and family, went out in nature, moved their bodies regularly, used social media less, and managed to get sufficient sleep were more resilient throughout the COVID-19 pandemic. We believe that these factors should be central to decision-makers’ efforts to support adolescents as they attend to their health needs as our society continues to experience the effects of the pandemic and faces future crises.

## Supplementary Information

**Additional file 1:** **Supplementary Text 1.** Summary of Psychometrically Validated Scales. **Supplementary Text 2. **Summary of Scales Developed by McKerracher et al.

## Data Availability

Custom scripts for quantitative analyses are available from the GitHub repository at https://github.com/kennek6/Cantec19. Anonymized and cleaned quantitative data used in these analyses will also be archived on GitHub if/when this manuscript is accepted for publication, and before it is published online. Anonymised qualitative data in the form of open-ended survey responses decoupled from other survey responses, as well as de-identified FGD notes/ summary schemes will be shared with researchers who provide a methodologically sound proposal. Proposals should be directed to luseadramckerracher@aias.au.dk; to gain access to qualitative data, data requestors will need to sign a data access agreement.

## Abbreviations

WHO: World Health Organization
COVID-19: Coronavirus disease-19
SARS-CoV-2: Severe acute respiratory syndrome coronavirus-2
CANTEC-19: CANadian Teens Experiencing COVID-19
FGD: Focus group discussion
FFQ: Food frequency questionnaire
FAMD: Factor analysis of mixed data
FA1: First factor
NB: Non-binary
