# Supplementary material for: Resilience in adolescence during the COVID-19 crisis in Canada
Source: BMC Public Health. 2023 Jun 6;23:1097. doi: 10.1186/s12889-023-15813-6 (PMC10243272; doi:10.1186/s12889-023-15813-6)
Supplement: Supplementary file 1 — Additional file 1: Supplementary Text 1. Summary of Psychometrically Validated Scales. Supplementary Text 2. Summary of Scales Developed by McKerracher et al. [file 12889_2023_15813_MOESM1_ESM.docx]

**SUPPLEMENTARY**

**Supplementary Text 1: Summary of Psychometrically Validated Scales**

| **Scale** | **Scale Description** | **Response Options** | **Scoring** |
| --- | --- | --- | --- |
| Mental Health & Wellbeing |  |  |  |
| Cantril Ladder | Two scales measured quality of life before the start of the COVID-19 pandemic lockdown and quality of life over the past two weeks. | 1 (worst possible life for you) to 10 (best possible life for you) | Higher scores represent higher quality of life. |
| Short Warwick-Edinburgh Mental Wellbeing Scale | 7-item measure of mental wellbeing that focuses on feelings and thoughts in the last two weeks (e.g. “I’ve been feeling relaxed”). | None of the time (1), Rarely (2), Some of the time (3), Often (4), All of the time (5) | Higher scores represent positive subjective mental wellbeing. |
| Child Health Utility 9D Questionnaire | 9-item paediatric measure of quality of life on the day of completing the questionnaire. The nine dimensions measured include: “Worried”, “Sad”, “Pain”, “Tired”, “Annoyed”, “Schoolwork/homework”, “Sleep”, “Daily Routine”, and “Able to join activities”. | There are 5 response levels for each dimension; for example, I don’t feel worried today (1), I feel a little bit worried today (2), I feel a bit worried today (3), I feel quite worried today (4), I feel very worried today (5). | A higher score represents lower subjective quality of life within a particular dimension. |
| Diet & Food Security |  |  |  |
| PrimeScreen Food Frequency Questionnaire | 18-item measure of food item consumption frequency (e.g. eggs, processed meats, baked products). | Less than once a week (1), Once a week (2), 2-4 times a week (3), Every day, or pretty much every day (4), Twice or more a day (5) | A high item score represents high consumption of a particular food type. |
| Hunger Vital Sign™ | 2-item measure of food insecurity in the past 12 months: “…did you or did anyone in your household worry whether your food would run out before you got money to buy more?” and “…did you ever find that the food in your household just didn’t last and you didn’t have money to get more?” | Often true (1), Sometimes true (2), Never true (3) | Higher scores represent greater food security. |

**Supplementary Text 2. Summary of Scales Developed by McKerracher et al.**

| Other Teen Health Behaviours |  |  |  |
| --- | --- | --- | --- |
| Sleep during the COVID-19 pandemic | 8-item measure of sleep quality and habits on the day of completing the questionnaire, in the past 6 months, and compared to before the COVID-19 pandemic. | Items have between 5 and 7 response levels. For example:  In general, I think I sleep _______ than I did before the  start of COVID-19. Response options include: A lot more (1), A little more (2), About the same (3), A little less (4), A lot less (5). | A higher score on sleep and waking time items represents a later sleep and waking time. A higher score on items about dysfunctional sleep represents higher sleep dysfunction. The exception is the last item for which a low score (A lot more) or a high score (A lot less) may indicate high sleep dysfunction. See the last item under “Response Options” column. |
| Physical activity during the COVID-19 pandemic | 7-item measure of time spent doing physical activity yesterday, in the past 6 months, and compared to before the COVID-19 pandemic. | Items have between 4 and 6 response levels. For example: In general, I think I spend _______ time on physical activities like walking, running, playing sports, doing workouts, helping with chores now compared to what I did before COVID-19. Response options include: A lot more (1), A little bit more (2), About the same (3), A little bit less (4), A lot less (5). | A higher score on the first six items represents higher activity levels. The exception is the last item for which a higher score represents less physical activity. See the last item under “Response Options” column. |
| Personal care during the COVID-19 pandemic | 2-item measure of time spent doing personal care activities (e.g. showering, dressing, hair care, nail care, skin care, make up, etc.) in the past 6 months and compared to before the COVID-19 pandemic. | Items include:  “Thinking about my life over the last 6 months, I usually spend _____ mins per day on showering, dressing, hair care, nail care, skin care, make up, etc.:”. Response options include: Fewer than 15 (1), 15-30 (2), 30-60 (3), 60-90 (4), 90+ (5)  “In general, I think I spend _____ time now on personal care activities than I did before COVID-19”. Response options include: A lot more (1), A little bit more (2), About the same (3), A little bit less (4), A lot less (5). | A higher score on the first item represents more time spent on personal care. A higher score on the second item represents less time spent on personal care. |
| Hand hygiene during the COVID-19 pandemic | 2-item measure of time spent washing or sanitizing hands in the past 6 months and compared to before the COVID-19 pandemic. | Items include:  “Thinking about my life over the last 6 months, I wash my hands thoroughly with soap or use hand sanitizer…”. Response options include: Fewer than 3 times a day (1), 3-5 times per day (2), 6-10 times per day (3), More than 10 times per day (4), It really depends on whether I am at home all day or out for work/ school/ socializing. Frankly, this question is kind of hard to answer (5).  “In general, I think I wash/sanitize my hands _____ frequently than I did before COVID-19”. Response options include: A lot more (1), A little bit more (2), About the same (3), A little bit less (4), A lot less (5). | A higher score on the first item generally represents more time spent engaging in hand hygiene activities. However, the highest response (5) does not necessarily indicate a lot of time spent engaging in hand hygiene activities. A higher score on the second item represents less time spent engaging in hand hygiene. |
| Screen time during the COVID-19 pandemic | 5-item measure of screen time during the past 6 months and compared to before the COVID-19 pandemic. | Items have between 5 and 6 response levels. For example, “In general, I think I spend _____ time using screens for entertainment or distraction than I did before the COVID-19 pandemic”. Response options include: A lot more (1), A little bit more (2), About the same (3), A little bit less (4), A lot less (5). | A higher score on the first four items represents more screen time. The exception is the last item for which a higher score represents less screen time. See the last item under “Response Options” column. |
| Work, volunteerism, studying, and leadership activities during the COVID-19 pandemic | 4-item measure of time spent working, volunteering, studying, and engaging in leadership activities during the past 6 months. | The first three items have 6 response levels: Never (1), Once every few weeks (2), About once a week (3), A few times a week (4), Every day or almost every day (5), More than once a day (6). The fourth item has four 4 response levels (“…I usually do school work for at least 10 hours per week”): Never (1), Once every few weeks (2), Most weeks (3), Every week (4). | Higher scores represent more time spent working, volunteering, studying, and/or engaging in leadership activities. |
| Miscellaneous leisure activities during the COVID-19 pandemic | 14-item measure of time spent engaging in miscellaneous leisure activities (e.g. art, creative writing, hanging out with friends, reading books/magazines). | Never (1), Once every few weeks (2), About once a week (3), A few times a week (4), Every day or almost every day (5), More than once a day (6) | A high score represents more time spent engaging in a particular activity. |
